# Characterization of Two Novel AmpC Beta-Lactamases from the Emerging Opportunistic Pathogen, *Cedecea neteri*

**DOI:** 10.3390/antibiotics12020219

**Published:** 2023-01-20

**Authors:** Stephen M. Sharkady, Brandon Bailey, Dorothea K. Thompson

**Affiliations:** Department of Pharmaceutical and Clinical Sciences, College of Pharmacy & Health Sciences, Campbell University, Buies Creek, NC 27506, USA

**Keywords:** *Cedecea neteri*, opportunistic pathogen, antibiotic resistance, AmpC, beta-lactamase, ESAC, nitrocefin, CNE-1, CNE-2

## Abstract

The genus *Cedecea* (family *Enterobacteriaceae*) causes a wide spectrum of acute infections in immunocompromised hosts, from pneumonia and bacteremia to oral ulcers and dialysis-related peritonitis. While *Cedecea* infections are reported infrequently in the literature, documented clinical cases of this emerging opportunistic human pathogen have occurred worldwide. *Cedecea neteri* has clinical significance and exhibits antimicrobial drug resistance. However, little is known about the molecular basis underlying the resistance phenotypes in *C. neteri*. We previously hypothesized that the open-reading frame *cnt10470* in the *C. neteri* SSMD04 genome encodes a chromosomal Ambler class C (AmpC) β-lactamase based on sequence homology. In this study, recombinant polyhistidine-tagged proteins were created by cloning the putative *ampC* genes from SSMD04 and *C. neteri* ATCC 33855 (a clinical isolate) into the pET-6xHN expression vector, overexpressing the proteins, and then purifying the recombinant AmpCs (rAmpCs) using immobilized metal affinity chromatography (Ni-NTA). The in vitro enzymatic analysis of the purified rAmpCs was performed to determine the *K_m_* and *k_cat_* for various β-lactam substrates. The rAmpCs are functional class C β-lactamases when assayed using the chromogenic β-lactamase substrate, nitrocefin. The presence of functional AmpCs in both *C. neteri* strains underscores the necessity of performing antibiotic susceptibility testing in the management of *C. neteri* infections.

## 1. Introduction

The *Cedecea* genus consists of facultative anaerobic, gram-negative bacilli that belong to the *Enterobacteriaceae* family [1]. Initially isolated at the US Centers for Disease Control and Prevention (CDC) in 1981, *Cedecea* (formerly the CDC enteric group 15) currently comprises three validly named species: *Cedecea neteri*, *Cedecea lapagei*, and *Cedecea davisae* [2]. Although *Cedecea* species have not been documented to cause invasive infections in healthy individuals, all three species were reported to cause bacteremia and pneumonia in severely immunocompromised patients (reviewed in [3]), thus promoting the recognition of these bacteria as emerging opportunistic pathogens. Clinical cases of *Cedecea* infections, albeit rare, have been described worldwide and may be underreported. We previously detailed the first case of urinary catheter colonization by multidrug-resistant *C. neteri* isolated from an elderly male patient with benign prostatic hyperplasia and stage three chronic kidney disease [4]. Most recently, *C. neteri* was attributed to the etiology of a urinary tract infection in a pregnant woman with polyhydramnios [5]. This *C. neteri* isolate was resistant to most third- and fourth-generation cephalosporins, with the exception of a few amino- and carboxy-penicillins. While patients generally recover with timely and appropriate antibiotic therapy, fatal outcomes have resulted from *Cedecea* infections [6,7], underscoring the need for the effective therapeutic management of these infections.

An increasing prevalence of multidrug resistance among bacterial pathogens in both hospital and community settings presents a serious challenge to successful therapeutic intervention, particularly in critically ill patients and those with multiple comorbidities. The rise of antibiotic-resistant gram-negative infections is strongly correlated with the production of β-lactamases, enzymes that hydrolyze the amide bond of the four-membered critical β-lactam ring, thereby inactivating the antibacterial efficacy of β-lactam drugs [8]. Beta-lactamases comprise a large heterogeneous group of enzymes that exhibit a range of biochemical and hydrolytic properties [9,10,11]. The Ambler classification scheme distributes β-lactamases into four classes (A, B, C, and D) according to their primary amino acid sequence homology [10]. One of the most clinically relevant branches of the β-lactamase lineage in *Enterobacteriaceae* is the active site serine class C β-lactamases or AmpC enzymes, which confer bacterial resistance to penicillins and cephalosporins [12]. A novel AmpC β-lactamase (CDA-1) was characterized from a carbapenem-resistant *C. davisae* clinical isolate with catalytic properties resembling those of a chromosome-borne β-lactamase from *Enterobacter cloacae* [13]. AmpC production, in combination with porin deficiency, accounted for the carbapenem resistance of this *C. davisae* isolate. Of particular significance, a recent case reported the development of AmpC hyperproduction in a *C. davisae* infection associated with a complex hand trauma involving skin and soft tissue necrosis, surgical debridement, and initial treatment with amoxicillin/clavulanate [14]. The authors found that the evolution of in vivo resistance in *C. davisae* was due to a mutation leading to a His75Arg alteration in *ampD*, part of the transcriptional regulatory system for inducible *ampC* expression. The presence of multiple β-lactamase genes in the sequenced genomes of the environmental isolates of *C. neteri* was noted previously, but the functionality of the encoded proteins was not determined [4,15].

Despite increasing reports of the clinical association of *Cedecea* species with infections in immunocompromised patients, very little is known about the molecular mechanisms underlying the antibiotic resistance phenotypes documented for these organisms. Knowledge of the genetic and biochemical basis of drug resistance in emerging opportunistic pathogens, including *Cedecea* spp., is needed to inform the therapeutic management, particularly the empirical treatment, of such infections. The present work details the expression, purification, and catalytic properties of an AmpC from an environmental and clinical isolate of *C. neteri*.

## 2. Results

### 2.1. Sequence Analysis of AmpC Proteins

**Figure 1:**
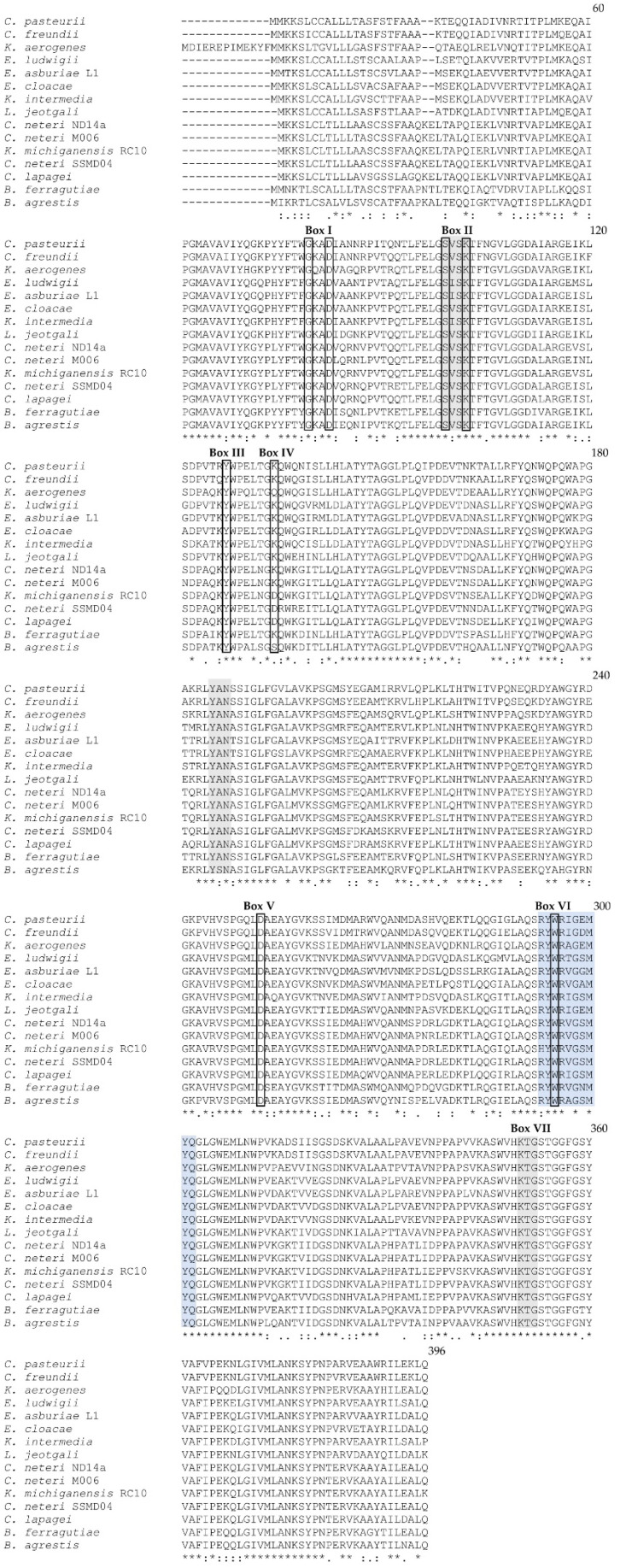
ClustalW multiple alignments of amino acid sequences of class C β-lactamases with annotated β-lactamases from three *C. neteri* environmental isolates (SSMD04 JT31_10470, ND14a LH86_07210, and M006 LH23_07270) and *C. lapagei*. The conserved sequence motifs specific for Ambler class C β-lactamases (AmpCs) are shaded in gray. The specific fingerprint sequence conserved among CMY family class C β-lactamases, as described in [20], is presented in light blue shading. The conserved residues corresponding to boxes I–VII in Joris et al. [18] are indicated. The active site serine residue is located in box II within the conserved S-X-SK motif. The AmpC primary sequences are from the following organisms: *Citrobacter pasteurii* UMH17 (GenBank accession no. CP024676.1), *Citrobacter freundii* complex sp. CFNIH3 (GenBank accession no. CP026235.1), *Klebsiella aerogenes* EA1509E (GenBank accession no. FO203354.1), *Enterobacter ludwigii* EcWSU1 (GenBank accession no. CP002886.1), *Enterobacter asburiae* L1 (GenBank accession no. CP007546.1), *Enterobacter cloacae* subsp. cloacae ATCC 13047 (GenBank accession no. CP001918.1)(CMH-4), *Kluyvera intermedia* (GenBank accession no. LR134138.1), *Lelliottia jeotgali* PFL01 (GenBank accession no. CP018628.1), *Cedecea neteri* ND14a (GenBank accession no. CP009459.1), *Cedecea neteri* M006 (GenBank accession no. CP009458.1), *Klebsiella michiganensis* RC10 (GenBank accession no. CP011077.1 (Anomalous assembly)), *Cedecea neteri* SSMD04 (GenBank accession no. CP009451.1), *Cedecea lapagei* NCTC11466 (GenBank accession no. LR134201.1), *Buttiauxella ferragutiae* H4-C11 (GenBank accession no. CP093332.1), and *Buttiauxella agrestis* DSM9389 (GenBank accession no. AP023184.1). Consensus symbols below the alignments are defined as follows: “*” (asterisk) indicates amino acid positions that have a single, fully conserved residue (i.e., identical residue); “:” (colon) indicates conservation between amino acids of strongly similar properties; and “.” (period) indicates conservation between amino acids of weakly similar properties.

Previously, we hypothesized that ORF cnt10470 in the *C. neteri* SSMD04 genome encodes a chromosome-borne Ambler class C β-lactamase or AmpC [4]. Based on its nucleotide sequence [16], the cnt10470 gene is predicted to encode a 382 amino acid protein with a calculated molecular mass of 42.2 kDa. The comparative multiple sequence analysis using ClustalW [17] revealed that Cnt10470 from SSMD04 shares a high sequence identity with AmpC from *Buttiauxella ferragutiae* (77%), *Enterobacter cloacae* (75%), and a CMY family class C β-lactamase from *Citrobacter freundii* (74%) (Figure 1). Besides the orthologs in the closely related *C. neteri* strains ND14a and M006, SSMD04 Cnt10470 exhibited the highest sequence identity with a β-lactamase from *Klebsiella michiganensis* RC10 (93%), although it must be stated that this was labeled as an anomalous genome (possible reasons for this discrepancy may be found at www.ncbi.nlm.nih.gov/assembly/help/anomnotrefseq/ (accessed on 4 January 2023). The deduced Cnt10470 protein contains the three conserved signature motifs characteristic of class C β-lactamases, including the key active site serine residue [18,19]. The active site serine residue occurs in the first position of a conserved tetrad (S-X-S-K) located near the *N*-terminus of Cnt10470 (Figure 1: Box II). Similar to *Streptomyces* R61 DD-peptidase/PBP, a conserved triad (K-T-G) occurs approximately 47 residues upstream of the *C*-terminus of Cnt10470 (Figure 1: Box VII). The comparative sequence analysis also revealed additional conserved residues described as boxes I-VII in [18]. The putative AmpC of *C. neteri* SSMD04 contains a fingerprint sequence (RYWRxGxMYQ) specific for β-lactamases classified in the CMY family (see Figure 1: Box VI; [15,20]). The novel β-lactamases characterized in this study are named CNE-1 (*C. neteri* SSMD04) and CNE-2 (*C. neteri* ATCC 33855). The AmpC from *E. cloacae* (CMH-4) was selected for the kinetic study based on the identity similarity with the CNE-1 and CNE-2 β-lactamases (Figure 1), and it represents a frequently isolated organism from immunocompromised hospitalized patients with clinical infections [21].

### 2.2. Overexpression and Purification of rAmpC Proteins

The recombinant *AmpC* clones were used for the isolation of the recombinant proteins. To screen for the correct clones expressing the rAmpCs, colony PCR was performed using universal primers, the T7 promoter, and the T7 terminator and analyzed using agarose gel electrophoresis. The same colonies were cultured in duplicates in Luria-Bertani (LB)-ampicillin media. One culture was used to prepare the plasmid DNA, while the other was used to check for the expression of the rAmpC proteins. Briefly, a small culture was grown for ~12 h at 37 °C, at which time an aliquot was removed (no induction sample), and then protein expression was induced by the addition of IPTG to a final concentration of 0.1 mM. The culture was induced for 8 h, and a final aliquot was removed (+induction sample) for the analysis of the protein expression via SDS-PAGE. Three positive clones from each strain, *E. cloacae* ATCC 13047 (CMH-4), *C. neteri* SSMD04 (CNE-1), and *C. neteri* ATCC 33855 (CNE-2), were determined using colony PCR, and the proper protein expression of the rAmpCs was also confirmed with DNA sequencing.

**Figure 2:**
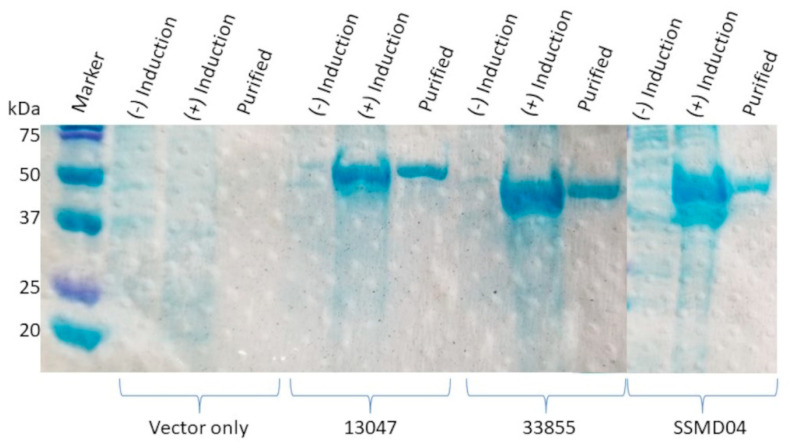
A 12% SDS-PAGE gel stained with Coomassie blue dye showing the purification progress and final purity of the various rAmpC proteins. The protein markers and sizes of the bands (kDa) are shown on the left. The four sets of samples are from *E. coli* BL21/DE3 cells containing the vector only (pET6xHN-N) and rAmpCs from *E. cloacae* 13047, *C. neteri* 33855, and *C. neteri* SSMD04. Each lane contains ~20 µg of total protein as determined with a BCA assay from the lysate minus induction, the lysate plus induction, and purified (after dialysis). The predicted sizes of the rAmpCs are 50.8 kDa, 45.1 kDa, and 45.4 kDa for CMH-4, CNE-2, and CNE-1, respectively.

Initially, the rAmpC proteins were produced from 500 mL cultures grown in LB with ampicillin at 37 °C until the OD_600_ was ~0.4, at which time IPTG was added to a final concentration of 0.1 mM to induce protein expression. Then, the cultures were grown at various times (from 6 h to 16 h), either at 37 °C or 30 °C. The cells were harvested by centrifugation and resuspended in 300 mM sodium chloride, 50 mM sodium phosphate (pH 8), 10 mM imidazole, and 1 mM phenylmethylsulfonyl fluoride (PMSF). Lysis was performed by sonication, followed by the clarification of the lysate by centrifugation. After the purification of the rAmpC proteins under native conditions using nickel agarose resin, dialysis was performed (the buffer contained 50 mM sodium phosphate (pH 7), 200 mM sodium chloride, 0.5 mM β-mercaptoethanol, and 20% glycerol), which led to precipitation of the rAmpC proteins, leading to poor yields of the soluble final His-tagged rAmpC proteins. Although the yield was insufficient to perform all the kinetic assays, the rAmpC proteins were enzymatically active when tested with the fluorogenic β-lactam reporter substrate, nitrocefin (the activity and kinetic parameters of the rAmpC proteins are shown in Table 1). To overcome this problem, recombinant proteins were purified under denaturing conditions using 8 M urea and a low pH for elution from the nickel-agarose resin [22]. The renaturation of the rAmpCs was accomplished via serial dialysis from 6 M urea to 0 M urea using six different buffers for two hours each time. The final dialysis buffer contained 50 mM sodium phosphate (pH 7), 200 mM sodium chloride, 0.5 mM β-mercaptoethanol, and 20% glycerol. Figure 2 shows the results of the purification of the rAmpC proteins under denaturing conditions from the *E. cloacae*, SSMD04, and ATCC 33855 strains. The samples shown are minus induction (before the addition of IPTG), plus induction (12 h after the addition of IPTG), and the AmpC protein purified using nickel-agarose and dialyzed. The control samples used for the experiment were *E. coli* BL21/DE3 cells containing the empty vector, pET6xHN-N (Figure 2, vector only). The induction of the overexpression of the rAmpC proteins could be easily seen when comparing the minus (−) and plus (+) induction lanes. The observed sizes of the rAmpC proteins containing an N-terminal histidine tag align with the predicted values of 50.8 kDa, 45.1 kDa, and 45.4 kDa for 13047, 33855, and SSMD04, respectively (Figure 2).

### 2.3. Determination of Extended-Spectrum AmpC β-Lactamase (ESAC) and Class B Metallo-β-Lactamase Activity

**Figure 3:**
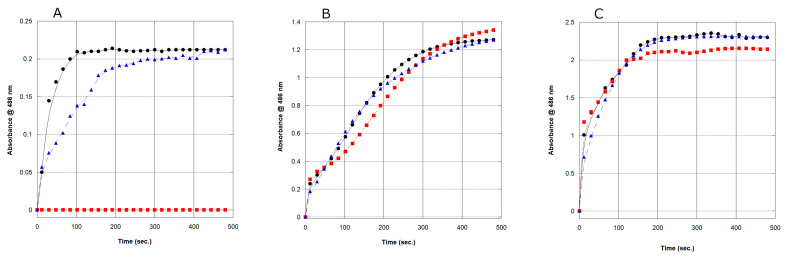
Hydrolysis of nitrocefin by the rAmpC proteins from *E. cloacae* 13047 (**A**), C. *neteri* 33855 (**B**), and *C. neteri* SSMD04 (**C**) in the absence (solid circle) and presence of either 2 mM clavulanic acid (red square) or 2 mM EDTA (blue triangle). For each panel, all reactions contained 500 nM rAMpC protein and 500 µM nitrocefin. Each data point represents the average of three experiments and the standard error was ≤5%.

We first confirmed that the β-lactamases studied here did not belong to the Ambler class B metallo-group, which requires Zn^2+^ for catalytic activity [23,24]. The reactions carried out in the absence and presence of EDTA with purified rAmpC enzymes using the reporter substrate, nitrocefin, were performed. The three rAmpC enzymes (CMH-4, CNE-1, and CNE-2) all hydrolyzed the substrate, nitrocefin, in the presence of EDTA, confirming that they did not belong to the Ambler class B metallo β-lactamases (Figure 3). The class C β-lactamases are functionally cephalosporinases that are generally resistant to clavulanic acid [25,26]. However, some AmpCs which show resistance to oxyimino-cephalosporins and are commonly sensitive to clavulanic acid were termed extended-spectrum AmpC β-lactamases (ESAC), similar to the extended-spectrum β-lactamases (ESBLs) of the A class according to the Bush-Jacoby classification [26,27,28]. Based on the sequence hallmarks of the predicted AmpCs (*C. neteri* SSMD04 and *C. neteri* 33855) and the known AmpC gene from *E. cloacae* 13047, we determined if any of these enzymes possessed ESAC activity, specifically relating to the inhibition with clavulanic acid. To test this aspect of ESAC activity, reactions were performed in the presence of clavulanic acid. Neither the *C. neteri* SSMD04, CNE-1 (Figure 3C), nor the *C. neteri* 33855, CNE-2 (Figure 3B), rAmpC was inhibited in the assays monitoring the cleavage of nitrocefin, but the AmpC from *E. cloacae* 13047, CMH-4, (Figure 3A) was completely inhibited by clavulanic acid, indicating it may possess ESAC activity. Further studies need to be performed with CMH-4 looking at the cleavage of various oxyimino-cephalosporins, such as ceftazidime and cefepime, to place it in the ESAC class definitively.

### 2.4. Enzymatic Activity and Kinetic Analysis of the rAmpC Proteins

The initial kinetic assays performed on all three purified rAmpC proteins with the chromogenic substrate, nitrocefin, were positive, confirming that the predicted ORF cnt10470 in *C. neteri* SSMD04 is a functional β-lactamase. To define the enzymatic parameters of the rAmpCs, a kinetic analysis was performed. The kinetic parameters, *K_m_* and *k_cat_*, for the rAmpCs with the substrate nitrocefin were experimentally determined by performing a classic Michaelis-Menten enzyme analysis. Table 1 shows the results of monitoring the cleavage of the chromogenic substrate nitrocefin. The cleavage of the β-lactam ring of nitrocefin is observed as an increase in absorbance. The *K_m_* values were determined to be 11, 46, and 133 µM for CMH-4 from *E. cloacae* (ATCC 13047), CNE-1 from *C. neteri* (SSMD04), and CNE-2 from *C. neteri* (ATCC 33855), respectively. The rAmpCs from the *Cedecea* species showed a higher level of activity towards the cleavage of nitrocefin as defined by the higher *k_cat_* values (Table 1).

We then tried to determine the kinetic parameters for a number of antibiotics, including ampicillin, first- and second-generation cephalosporins, and imipenem. The cleavage of the antibiotics by the β-lactamases was monitored by following the decrease in absorbance correlating to the cleavage of the β-lactam ring using the specific molar extinction coefficients for the various antibiotics. Experimental parameters, such as temperature (up to 37 °C) and time courses for 24 h, were utilized in order to detect the direct cleavage of the substrates, but none were successful. To determine the *K_m_* of these poor substrates, kinetic assays were performed containing 200 µM nitrocefin as a good reporter substrate and 500 nM rAmpC in the presence of various concentrations of the poor substrate (i.e., the inhibitor). Lineweaver-Burk plots were utilized to determine the *K_I_* or *K_m, apparent_* for the poor substrates/antibiotics, and the results are summarized in Table 2.

All the rAmpCs bound well to the antibiotics (Table 2); however, in general, the rAmpC from *E. cloacae* bound most of the antibiotics with higher affinity than the rAmpCs from the *C. neteri* strains.

## 3. Discussion

This study shows that the chromosomally expressed ORF cnt10470 in the *C. neteri* SSMD04 genome encodes a functionally active Ambler class C β-lactamase, CNE-1, as well as the AmpC gene from *C. neteri* (ATCC 33855), CNE-2. When comparing the activity of CDA-1 (AmpC from *C. davisae*) to CNE-1 and CNE-2, the *K_m_* for imipenem was comparable at ~2 µM for all, whereas the observed *K_m_* for cefoxitin was 100-fold higher for the *neteri* enzymes compared to the one from *davisae* [13]. Although CNE-1 and CNE-2 are approximately 86% identical to CDA-1, there are differences between the genes (Supplemental Figure S1). CMH-4 has not been previously characterized but was chosen for this study based on its documented role as a frequent infectious agent in hospitalized patients who are immunocompromised. A closely related AmpC from *E. cloacae* P99 was characterized [29] and exhibited a similar *K_m_* for nitrocefin, but the *k_cat_* observed for CMH-4 in this study was ~100-fold less. One possible reason for the differences may be related to the way the enzymes were purified. For the previous studies, the AmpCs were purified under native conditions and some without a histidine tag, whereas we purified our lactamases under denaturing conditions, and they were re-folded.

To further characterize the rAmpCs in this study, we also tested to see if they exhibited extended-spectrum β-lactamase (ESBL) activity. ESBLs are characterized by their ability to hydrolyze third- and fourth-generation cephalosporins but are inhibited by clavulanic acid [25,30]. Most β-lactamases characterized as ESBLs belong to the Ambler class A enzymes found most commonly on extra-chromosomal plasmids; therefore, they are easily transferable to other bacteria, causing severe infections [8,24,25,31,32,33,34]. However, there are instances, although rare, of chromosomal Ambler class C cephalosporinases that also possess ESBL activity and are termed extended-spectrum AmpC β-lactamases (ESACs) [25,26,35]. The rAmpCs from both *C. neteri* strains tested were not inhibited by clavulanate and, thus, most likely, are not in the ESAC class. On the other hand, we discovered that the chromosomal rAmpC, CMH-4, from *E. cloacae* ATCC 13047 (GenBank accession no. ADF62789) was completely inhibited by clavulanate, indicating a possible novel ESAC not previously reported. *E. cloacae* ATCC 13047 in disk diffusion assays was shown to harbor extended-spectrum β-lactam activity, but this activity was attributed to a class A β-lactamase [31]. A protein alignment performed using CLUSTALW [www.genome.jp/tools-bin/clustalw (accessed on 2 December 2022)] between the CMH-4 from ATCC 13047 and two clinical strains (GC1 and CHE) from *E. cloacae* showed ~83% identity (Supplemental Figure S2), demonstrating that they are highly similar but different proteins. Several studies have investigated how specific protein mutations and natural variants of ESACs affect kinetic parameters, such as *K_m_* and *k_cat_* [12,36,37]. In future studies, it would be interesting to compare the AmpC from ATCC 13047 with other known ESAC variants.

In conclusion, the emerging opportunistic pathogen, *C. neteri*, which mainly causes clinical infections in immunocompromised patients, exhibited antimicrobial resistance to many first-line therapeutics [3]. To better understand and characterize the β-lactamase activity of a hypothetical AmpC in *C. neteri* SSMD04, we have cloned, purified, and biochemically characterized chromosomal AmpC genes from two other strains and shown all to be active Ambler type C enzymes. Additionally, we discovered that the chromosomal rAmpC, CMH-4, from *E. cloacae* ATCC 13047 (GenBank accession no. ADF62789) might possess ESAC activity since it is inhibited by clavulanic acid. The results here highlight the importance of understanding which β-lactamases are present and active within various strains and the importance of proper antimicrobial susceptibility testing prior to treatment, which would hopefully reduce the prevalence of antibiotic resistance in these strains in the future.

## 4. Materials and Methods

### 4.1. Bacterial Strains, Chemicals, and Supplies

The strains of *C. neteri* (ATCC 33855) and *E. cloacae* (ATCC 13047) were obtained from the American Type Culture Collection (ATCC). Stock solutions of ampicillin, cephalexin, imipenem, and penicillin were created using water, while cefazolin, cefoxitin, cefuroxime, and nitrocefin were dissolved in DMSO. All chemicals for this study were purchased from Sigma-Aldrich (St. Louis, MO, USA) unless otherwise specified.

### 4.2. Cloning of the AmpC Genes

All bacteria were grown in LB broth at 37 °C, and the genomic DNA was prepared from overnight cultures (Wizard Genomic Purification Kit, Promega, Madison, Wisconsin, USA). The predicted *AmpC* gene from *C. neteri* SSMD04 (GenBank accession no. CP009451.1(JT31_10470)) was synthesized by Twist Bioscience (South San Francisco, CA, USA). The known *AmpC* from *E. cloacae* ATCC 13047 (GenBank accession no. ADF62789) and predicted *AmpC*s from *C. neteri* SSMD04 and ATCC 33855 (RefSeq: GCF_001571265.1) were amplified using PCR from the genomic DNA or synthesized gene (*C. neteri* SSMD04) using the primer (Integrated DNA Technologies, Coralville, IA, USA) pairs below:

AmpC ATCC 13047

5′-TTCCACATAAGATCTCTATGAAAAACAAAACGTTAAACCGC-3′

5′-TATAGAACGTCTAGATTAGCGCAGCGCCTGTGCAATTGC-3′

AmpC ATCC 33855

5′-TTCCACATAAGATCTCTATGAAAAAATCCCTCTGCCTGACG-3′

5′-TATAGAACGTCTAGATTATTGCAGTGCTTCGAGAATAGCG-3′

AmpC SSMD04

5′-TTCCACATAAGATCTCTATTATGAAAAAATCCCTCTGCC-3′

5′-TATAGAACGTCTAGATTATTGCAGCGCTTCGAGAATAGC-3′

T7 Forward (promoter)

5′-TAATACGACTCACTATAGGG-3′

T7 Terminator

5′-GCTAGTTATTGCTCAGCGG-3′

The PCR-amplified genes and the expression vector, pET6xHN-N (Clontech Inc., San Jose, California, USA), were digested with *Bgl*II and *Xba*I (New England Biolabs, Ipswich, MA, USA) and then gel purified prior to ligation. Ligations were performed using T4 DNA ligase (New England Biolabs, Ipswich, Massachusetts, USA) and then transformed into *E. coli* BL21/DE3 calcium-competent cells. The positive clones were identified by performing colony PCR using the primers T7 forward and T7 terminator. The positive clones were confirmed using DNA sequencing (Eton Bioscience, Inc., Research Triangle, NC, USA).

### 4.3. Expression and Purification of AmpC Proteins

The expression vector pET6x-HN-N containing the recombinant *AmpC* (*rAmpC*) genes (designated by 13047 for *E. cloacae* ATCC 13047 or 33855 for *C. neteri* ATCC 33855 or SSMD04 for *C. neteri* SSMD04) was used to append a 6x histidine tag to the N-terminus of the rAmpC proteins, thereby facilitating purification using a Ni-agarose resin. The recombinant *AmpC* clones from *E. cloacae* ATCC 13047, *C. neteri* ATCC 33855, and *C. neteri* SSMD04 were grown in 200 mL cultures to an OD_600_~0.4. The expression was induced by the addition of isopropyl β-D-1-thiogalactopyranoside (IPTG) to a final concentration of 0.1 mM and grown for 16 h at 37 °C with shaking. The cells were harvested by centrifugation, and the rAmpC proteins were purified under denaturing conditions in a batch format using a Ni-agarose resin according to the manufacturer’s protocols (Qiagen, Hilden, Germany). The eluted rAmpC proteins were renatured using serial dialysis to remove all urea into a final buffer containing 50 mM sodium phosphate, 300 mM sodium chloride, and 20% glycerol (pH 7). The protein concentrations were determined using the BCA assay (Sigma-Aldrich, St. Louis, MO, USA) with bovine serum albumin as a standard [38]. The purification process and purity of the final fractions were analyzed using 12% tris-glycine SDS-PAGE gels (Bio-Rad Laboratories, Hercules, CA, USA). The SDS-PAGE gels were stained using Coomassie blue dye [39].

### 4.4. Kinetic Analysis of the rAmpC Proteins

The kinetic analysis was performed using the purified rAmpC proteins: *E. cloacae* 13047 as a positive control and the predicted AmpC proteins from *C. neteri* 33855 and SSMD04. The hydrolysis of substrates was monitored by the variation in absorbance due to the cleavage of the β-lactam ring in the molecules and correlated to the concentrations via the molar extinction coefficients. Each 200-µL reaction contained 500 nM of the enzyme (rAmpC), while the concentration of substrate was varied from 10 µM to 200 µM in the reaction buffer (50 mM sodium chloride, 50 mM sodium phosphate (pH 7)) at 30 °C. All reactions were run in triplicate using Corning^®^ UV-transparent 96 well plates (Sigma-Aldrich), and the reactions were initiated by the addition of the enzyme. The substrates used in this study were the following: nitrocefin (Δε_486_ = +15,000 M^−1^ cm^−1^) [40], ampicillin (Δε_235_ = −900 M^−1^ cm^−1^), cephalexin (Δε_260_ = −7000 M^−1^ cm^−1^), penicillin (Δε_240_ = −500 M^−1^ cm^−1^), cefazolin (Δε_320_ = 1067 M^−1^ cm^−1^), cefoxitin (Δε_265_ = −7000 M^−1^ cm^−1^), cefuroxime (Δε_262_ = −8450 M^−1^ cm^−1^), and imipenem (Δε_295_ = −9000 M^−1^ cm^−1^). The reaction progress was monitored using a BioTek Synergy H1 plate reader for 15 min at 486 nm for nitrocefin. For the substrate nitrocefin, the kinetic parameters *K_m_* and *k_cat_* were calculated by obtaining the initial velocities at specific substrate concentrations (three concentrations below and three concentrations above the *K_m_*) and then creating Michaelis-Menten (Equation (1)) plots. The kinetic parameters were determined by non-linear regression using Kaleidagraph version 4.5 (Synergy Software, Reading, PA, USA).

*V_0_* = V*_max_* x [S]/[S] + *K_m_* (1)

When the compounds tested acted as poor substrates or inhibitors of the enzymes, the reactions were performed in the presence of a good reporter substrate (nitrocefin). This allowed the determination of the *K_i_* or *K_m,apparent_* of the poor substrates/inhibitors. To determine the *K_m_* for ampicillin, cephalexin, penicillin, cefazolin, cefoxitin, cefuroxime, and imipenem, the *K_i_* was measured using reactions containing 500 nM rAmpC and 200 µM nitrocefin at various concentrations of the substrates/inhibitors. The initial velocities were obtained using non-linear regression and Equation (1), and then the Lineweaver-Burk plots (1/*v_0_* versus 1/[Substrate/Inhibitor]) were extrapolated using linear regression to determine the *K_i_* or *K_m,apparent_* for each substrate/inhibitor.

### 4.5. Assay for Extended-Spectrum AmpC β-Lactamase (ESAC) and Class B Metallo-β-Lactamase Activity

Similar to the kinetic analysis, 200-µL reactions contained 500 nM enzyme (rAmpC) and 500 µM nitrocefin in reaction buffer (50 mM sodium chloride, 50 mM sodium phosphate (pH 7)) at 30 °C. The reactions testing ESBL activity (related to the observation that most ESBL enzymes are inhibited by clavulanic acid) were performed in the absence or presence of 2 mM clavulanic acid [25,41]. Class B β-Lactamase activity was tested by reactions performed in the absence or presence of 2 mM ethylenediaminetetraacetic acid (EDTA) [42]. All reactions were performed in triplicate, and nitrocefin cleavage was monitored at 486 nm for 8 min.

## Figures and Tables

**Table 1:** Kinetic parameters (*K_m_* and *k_cat_*) of the purified rAmpC enzymes from *E. cloacae* (ATCC 13047), *C. neteri* (SSMD04), and *C. neteri* (ATCC 33855) using nitrocefin as the substrate.

Strain	*K_m_* (µM)	*k_cat_* (s^−1^)	*k_cat_*/*K_m_* (mM^−1^s^−1^)
*E. cloacae* (ATCC 13047)	11 ± 2	0.83 ± 0.04	7.5
*C. neteri* (SSMD04)	46 ± 5	1.93 ± 0.16	41.9
*C. neteri* (ATCC 33855)	133 ± 11	3.13 ± 0.27	23.5

**Table 2:** ***K_m,apparent_*** of the poor substrates for the purified rAmpC enzymes from *E. cloacae* (ATCC 13047), *C. neteri* (SSMD04), and *C. neteri* (ATCC 33855) using nitrocefin as a reporter substrate.

		*K_m,apparent_* (µM) ^1^	
	**ATCC 13047**	**SSMD04**	**ATCC 33855**
**Antibiotic**			
Ampicillin	7	72	12
Penicillin	33	58	11
Cefazolin	0.43	67	100
Cephalexin	6.8	2	5
Cefoxitin	12	48	54
Cefuroxime	0.35	5	4
Imipenem	0.85	5	2

^1^ All standard deviations were ≤15%.

## Data Availability

Data is contained within the article and supplementary material.

## Supplementary Materials

The following supporting information can be downloaded at: https://www.mdpi.com/article/10.3390/antibiotics12020219/s1, Supplemental Figure S1: ClustalW multiple sequence alignment of Cedecea AmpCs and CMH-4 from E. cloacae. CDA-1, CNE-1, CNE-2, CMH-4 from E. cloacae ATCC 13047, and C. lapagei AmpC; Supplemental Figure S2: ClustalW multiple sequence alignment of E. cloacae species AmpCs. CMH-4 from E. cloacae ATCC 13047, GC1 and CHE.
