# Supplementary material for: Characterization of Two Novel AmpC Beta-Lactamases from the Emerging Opportunistic Pathogen, Cedecea neteri
Source: Antibiotics (Basel). 2023 Jan 20;12(2):219. doi: 10.3390/antibiotics12020219 (PMC9952435; doi:10.3390/antibiotics12020219)
Supplement: Supplementary file 1 [file antibiotics-12-00219-s001.zip › antibiotics-2125776-supplementary.pdf]

Supplemental Figure S1

```
CLUSTAL 2.1 Multiple Sequence Alignments


Sequence type explicitly set to Protein
Sequence format is Pearson
Sequence 1: CMH-4        381 aa
Sequence 2: CNE-1        382 aa
Sequence 3: CNE-2        382 aa
Sequence 4: CDA-1        382 aa
Sequence 5: C_lapagei    380 aa
Start of Pairwise alignments
Aligning...



Sequences (1:2) Aligned. Score: 74.0157
Sequences (1:3) Aligned. Score: 73.4908
Sequences (1:4) Aligned. Score: 74.2782
Sequences (1:5) Aligned. Score: 78.9474
Sequences (2:3) Aligned. Score: 93.4555
Sequences (2:4) Aligned. Score: 86.1257
Sequences (2:5) Aligned. Score: 74.2105
Sequences (3:4) Aligned. Score: 86.3874
Sequences (3:5) Aligned. Score: 74.4737
Sequences (4:5) Aligned. Score: 77.8947
Guide tree file created:   [clustalw.dnd]

There are 4 groups
Start of Multiple Alignment

Aligning...
Group 1: Sequences:   2      Score:6105
Group 2: Sequences:   3      Score:5904
Group 3: Sequences:   2      Score:6278
Group 4: Sequences:   5      Score:5955
Alignment Score 19417

CLUSTAL-Alignment file created  [clustalw.aln]
```

---

clustalw.aln

```
CLUSTAL 2.1 multiple sequence alignment


CNE-1           -MKKSLCLTLLLAASCSSFAAQKELTAQQIEKLVNRTVAPLMKEQAIPGMAVAVIYKGYP
CNE-2           -MKKSLCLTLLLAASCSSLAAQKELTAQQIEKVVNRTVAPLMKEQAIPGMAVAVIYKGYP
CDA-1           -MKKSLCLTLLLAASCSTFAAPKQLTAQQIEKIVNRTISPLLKEQAIPGMAVAVIYKGYP
CMH-4           MMKKSLSCALLLSVACSAFAAP--MSEKQLADVVERTVTPLMKAQAIPGMAVAVIYQGQP
C_lapagei       -MKKSLCCALLLSASFSTLAAP--VTDKQLADIVNRTIAPLMQEQAIPGMAVAVIYQGKP
                 *****. :***:.: *::**   :: :*: .:*:**::**:: ************:* *

CNE-1           LYFTWGKADVQRNQPVTRETLFELGSVSKTFTGVLGGDALARGEISLSDPAQKYWPELTG
CNE-2           LYFTLGKADVQRNVPVTQQTLFELGSVSKTFTGVLGGDALARGEVSLSDPAQKYWPELNG
CDA-1           LYFTWGKADVQHNEPVTRQTLFELGSVSKTFTGVLGGDTLARGEISLSDPAQKYWPELTG
CMH-4           HYFTFGKADVAANKPVTPQTLFELGSVSKTFTGVLGGDAIARKEISLADPVTKYWPELTG
C_lapagei       HIFTWGKADIEANRPVTQQTLFELGSVSKTFTGVLGGDAIARGEIRLSDPVTKYWPELTG
                  ** ****:  * *** :*******************::** *: *:**. ******.*

CNE-1           DRWREITLLQLATYTAGGLPLQVPDEVTNNDALLKFYQTWQPQWAPGTQRLYANASIGLF
```

```
CNE-2           NQWKGITLLQLATYTTGGLPLQVPDEVTNSDELLKFYQDWQPQWAPGTQRLYANASIGLF
CDA-1           SQWKGITLLQLATYTAGGLPLQVPDEVTDSASLLNFYQSWQPQWAPGSKRLYANASIGLF
CMH-4           KQWQGIRLLDLATYTAGGLPLQVPDDVTDNASLLRFYQSWQPKWAPGTTRLYANTSIGLF
C_lapagei       KQWQHINLLHLATYTAGGLPLQVPDDVTDRAALLTFYQNWQPQWAPGEKRLYANASIGLF
                .:*: * **.*****:*********:**:   ** *** ***:****  *****:*****

CNE-1           GALMVKPSGMSFDKAMSKRVFEPLNLTHTWINVPATEESRYAWGYRDGKAVRVSPGMLDA
CNE-2           GALMVKPSGMSFEQAMSKRVFEPLNLTHTWIKVPATEESHYAWGYRDGKAVRVSPGMLDA
CDA-1           GALMVKPSGMGFEQAMTTRVLEPLKLAHTWITVPPAEESHYAWGYRNDKAVRVSPGMLDA
CMH-4           GSLAVKPSGMRFEQAMAERVFKPLKLNHTWINVPHAEEPHYAWGYREGKAVHVSPGMLDA
C_lapagei       GSLAVKPSGMSFEQAMTTRVLQPLKLNHTWLTVPESEQQHYAWGYRDGKAVHVSPGQLDA
                *:* ****** *::**: **::**:* ***:.** :*: :******:.***:**** ***

CNE-1           EAYGVKSSIEDMAHWVQANMAPDRLEDKTLQQGIRLAQSRYWRVGSMYQGLGWEMLNWPV
CNE-2           EAYGVKSSIEDMAHWVQANMAPHRVEDKTLAQGIQLAQSRYWRVGSMYQGLGWEMLNWPV
CDA-1           EAYGVKSSIEDMAHWVQANMVPERVEDQNLQQGIKLAQSRYWRIGSMYQGLGWEMLNWPL
CMH-4           EAYGVKSNVKDMASWVMANMAPETLPQSTLQQGIALAQSRYWRVGAMYQGLGWEMLNWPV
C_lapagei       EAYGVKTTIEDMASWVQANMNPATVSDQKLQQGITLAQSRYWQIGEMYQGLGWEMLNWPV
                ******:.::*** ** *** *  : :..* *** *******::* *************:

CNE-1           KGKTIIDGSDNKVALAPHPATLIDPPVPAVKASWVHKTGSTGGFGSYVAFIPEKQLGIVM
CNE-2           KGKTIIDGSDNKVALAPHPATLIDPPVTAVKASWVHKTGSTGGFGSYVAFIPEKQLGIVM
CDA-1           KGKVIIDGSDNKVALAPQTAVAIDPPAPLVKASWVHKTGSTGGFGSYVAFIPEKQLGIVM
CMH-4           DAKTVVDGSDNKVALAPLPVAEVNPPAPPVKASWVHKTGSTGGFGSYVAFIPEKQIGIVM
C_lapagei       KAKTVVTGSDNKVALAPATAVEINPPAPAVKASWVHKTGSTGGFGSYVAFIPQKDLGIVM
                ..*.:: ********** ... ::**.. ***********************:*::****

CNE-1           LANKSYPNTERVKAAYAILEALQ
CNE-2           LANKSYPNTERVKAAYAILEALQ
CDA-1           LANKSYPNPERVKAAYAILEALQ
CMH-4           LANKSYPNPVRVETAYRILDALQ
C_lapagei       LANKSYPNPARVDAAFRILDALQ
                ********. **.:*: **:***
```

**Supplemental Figure S1.** ClustalW (www.genome.jp/tools-bin/clustalw (Accessed 4 Jan. 2023) multiple sequence alignment of *Cedecea* AmpCs and CMH-4 from *E. cloacae*. CDA-1, CNE-1, CNE-2, CMH-4 from *E. cloacae* ATCC 13047, and *C. lapagei* AmpC.

## Supplemental Figure S2

```
CLUSTAL 2.1 Multiple Sequence Alignments


Sequence type explicitly set to Protein
Sequence format is Pearson
Sequence 1: GC1            384 aa
Sequence 2: CMH-4          381 aa
Sequence 3: CHE            375 aa
Start of Pairwise alignments
Aligning...



Sequences (1:2) Aligned. Score: 83.4646
Sequences (1:3) Aligned. Score: 97.8667
Sequences (2:3) Aligned. Score: 83.4667
Guide tree file created:   [clustalw.dnd]
```

There are 2 groups
Start of Multiple Alignment

Aligning...
Group 1: Sequences:   2      Score:6093
Group 2: Sequences:   3      Score:5760
Alignment Score 6454

CLUSTAL-Alignment file created  [clustalw.aln]

---

clustalw.aln

CLUSTAL 2.1 multiple sequence alignment

```
GC1             MMKKSLCCALLLGISCSALATPVSEKQLAEVVANTVTPLMKAQSVPGMAVAVIYQGKPHY
CHE             MMKKSLCCALLLGISCSALATPVSEKQLAEVVANTVTPLMKAQSVPGMAVAVIYQGKPHY
CMH-4           MMKKSLSCALLLSVACSAFAAPMSEKQLADVVERTVTPLMKAQAIPGMAVAVIYQGQPHY
                ******.*****.::***:*:*:******:** .*********::***********:***

GC1             YTFGKADIAANKPVTPQTLFELGSISKTFTGVLGGDAIARGEISLDDPVTRYWPQLTGKQ
CHE             YTFGKADIAANKPVTPQTLFELGSISKTFTGVLGGDAIARGEISLDDPVTRYWPQLTGKQ
CMH-4           FTFGKADVAANKPVTPQTLFELGSVSKTFTGVLGGDAIARKEISLADPVTKYWPELTGKQ
                :******:****************:*************** **** ****:***:*****

GC1             WQGIRMLDLATYTAGGLPLQVPDEVTDNASLLAFYQNWQPQWKPGTTRLYANASIGLFGA
CHE             WQGIRMLDLATYTAGGLPLQVPDEVTDNASLLRFYQNWQPQWKPGTTRLYANASIGLFGA
CMH-4           WQGIRLLDLATYTAGGLPLQVPDDVTDNASLLRFYQSWQPKWAPGTTRLYANTSIGLFGS
                *****:*****************:******** ***.***:* *********:******:

GC1             LAVKPSGMPYEQAMTTRVLKPLKLDHTWINVPKAEEAHYAWGYRDGKAVRAVRVSPGMLD
CHE             LAVKPSGMPYEQAMTTRVLKPLKLDHTWINVPKAEEAHYAWGYRDG---KAVRVSPGMLD
CMH-4           LAVKPSGMRFEQAMAERVFKPLKLNHTWINVPHAEEPHYAWGYREG---KAVHVSPGMLD
                ******** :****: **:*****:*******:***.*******:*   :**:*******

GC1             AQAYGVKTNVQDMANWVMANMAPENVADASLKQGIALAQSRYWRIGSMYQGLGWEMLNWP
CHE             AQAYGVKTNVQDMANWVMANMAPENVADASLKQGIALAQSRYWRIGSMYQGLGWEMLNWP
CMH-4           AEAYGVKSNVKDMASWVMANMAPETLPQSTLQQGIALAQSRYWRVGAMYQGLGWEMLNWP
                *:*****:**:***.*********.:.:::*:************:*:*************

GC1             VEANTVVEGSDSKVALAPLPVAEVNPPAPPVKASWVHKTGSTGGFGSYVAFIPEKQIGIV
CHE             VEANTVVEGSD------PLPVVEVNPPAPPVKASWVHKTGSTGGFGSYVAFIPEKQIGIV
CMH-4           VDAKTVVDGSDNKVALAPLPVAEVNPPAPPVKASWVHKTGSTGGFGSYVAFIPEKQIGIV
                *:*:***:***      ****.**************************************

GC1             MLANTSYPNPARVEAAYHILEALQ
CHE             MLANTSYPNPARVEAAYHILEALQ
CMH-4           MLANKSYPNPVRVETAYRILDALQ
                ****.*****.***:**:**:***
```

**Supplemental Figure S2.** ClustalW(www.genome.jp/tools-bin/clustalw (Accessed 2 Dec. 2022) multiple sequence alignment of *E. cloacae* species AmpCs. CMH-4 from *E. cloacae* ATCC 13047, GC1 and CHE.
